# Improved energy efficiency in microbial fuel cells by bioethanol and electricity co-generation

**DOI:** 10.1186/s13068-022-02180-4

**Published:** 2022-08-17

**Authors:** Rong Xie, Shuang Wang, Kai Wang, Meng Wang, Biqiang Chen, Zheng Wang, Tianwei Tan

**Affiliations:** National Energy R&D Center for Biorefinery, Beijing Key Lab of Bioprocess, College of Life Science and Technology, Beijing University of Chemical Technology, No. 15 North 3rd Ring Rd East, Beijing, 100029 People’s Republic of China

**Keywords:** Microbial fuel cells, *Saccharomyces cerevisiae*, Ethanol, Electricity

## Abstract

**Background:**

Microbial electricity production has received considerable attention from researchers due to its environmental friendliness and low price. The increase in the number of intracellular electrons in a microbial fuel cell (MFC) helps to improve the MFC performance.

**Results:**

In this study, we accumulated excess electrons intracellularly by knocking out the gene related to intracellular electron consumption in *Saccharomyces cerevisiae*, and the elevated intracellular electron pool positively influenced the performances of MFCs in terms of electricity production, while helping to increase ethanol production and achieve ethanol and electricity co-production, which in turn improved the utilization of substrates. The final knockout strain reached a maximum ethanol yield of 7.71 g/L and a maximum power density of 240 mW/m^2^ in the MFC, which was 12 times higher than that of the control bacteria, with a 17.3% increase in energy utilization.

**Conclusions:**

The knockdown of intracellular electron-consuming genes reported here allowed the accumulation of excess electrons in cells, and the elevated intracellular electron pool positively influenced the electrical production performance of the MFC. Furthermore, by knocking out the intracellular metabolic pathway, the yield of ethanol could be increased, and co-production of ethanol and electricity could be achieved. Thus, the MFC improved the utilization of the substrate.

**Supplementary Information:**

The online version contains supplementary material available at 10.1186/s13068-022-02180-4.

## Background

The use of fossil energy leads not only to global climate change, but also to an increase in energy shortages due to the non-renewable nature of fossil energy [1]. Renewable energy use can help solve the current problems of energy shortages and inadequate power supplies, as well as achieve sustainable development, efficient use, and resource conservation of energy [2]. As one of the most promising biofuels to replace fossil fuels, bioethanol has the advantage of being widely used and derived from renewable products (e.g., biomass such as straw) [3]. Microorganisms can produce ethanol from raw materials such as molasses and cellulose under the action of fermentation. As a microorganism suitable for industrial production, brewer's yeast has a long history of fermenting and producing ethanol, and it is easy to cultivate, fast growing and metabolism, simple and cheap, safe and highly tolerant of ethanol [4]. Under anaerobic conditions, 1 mol of glucose will produce 2 mol of pyruvate. Pyruvate will be catalyzed by pyruvate decarboxylase and is further oxidized to 2 mol acetaldehyde and 2 mol carbon dioxide is produced. Next, 2 mol of acetaldehyde is passed through ethanol dehydrogenase to produce 2 mol of ethanol. Since ethanol dehydrogenase is an NADH-dependent enzyme, the intracellular accumulation of NADH is able to increase ethanol production to some extent [5, 6]. In addition, the fermentation of organic matter by brewer's yeast to produce ethanol is accompanied by the oxidation of large amounts of NADH to NAD^+^.

The intracellular reduced coenzyme NADH or NADPH can be collected in the form of electrons for power generation. Microbial devices that use organic or inorganic feedstocks to generate electricity are often called microbial fuel cells (MFCs). In microbial fuel cells, microorganisms transfer electrons to solid electrodes. Anode microbial fuel cells can be divided into two categories based on microbial utilization. Fuel cells in the first category utilize a single compound, such as glucose [7, 8], xylose [9], cellulose [10], or acetate [11], and use a single strain of bacteria as the inoculum source for MFCs. Fuel cells in the second category usually use waste with a complex composition, such as domestic sewage [12], industrial wastewater [13], medical wastewater [14], and other types of wastewater (e.g., straw hydrolysate, human excreta) [15–18], and use colonies in the sludge as a source of inoculum for MFC. Microorganisms in the anode transfer electrons to the electrode, while microorganisms from the cathode can catalyze oxygen reduction reactions instead of inorganic catalysts, receiving electrons [19, 20]. Some microorganisms are attached to the electrode, and intracellular electrons can be transferred directly to the electrode, i.e., direct electron transfer (DET), while microorganisms free in the medium need an electron transfer medium to achieve electron transfer [21, 22]. In microbial fuel cells, the electron transfer efficiency is improved by adding redox mediators, genetic engineering methods to modify microorganisms, using materials with better electrical conductivity as electrodes, and so on. For example, Lithuania et al. summarized the biocompatibility of conducting polymers, which are commonly used in biofuel cells to improve the electron transfer efficiency [23, 24]. In addition, two-dimensional materials such as MXenes are often used in the design of biosensors and biofuel cells to improve the electron transfer efficiency [25].

Due to the partial energy consumption of *Saccharomyces cerevisiae* during the fermentation for ethanol production, the energy utilization is reduced. When microbial fuel cells use substrates to generate electricity, the substrates have less application in the production of the product. Thus, by culturing *Saccharomyces cerevisiae* in a microbial fuel cell anode chamber, on the one hand, the microorganisms can use the substrate for fermentation to produce ethanol. On the other hand, electrons generated from the NADH/NAD^+^, NADPH/NADP^+^ redox cycle can be extracted for power generation by using MFC technology. Thus, by harvesting some of the energy that would otherwise be lost during the fermentation process in the form of electrical energy, the utilization of the substrate is improved.

**Fig. 1:**
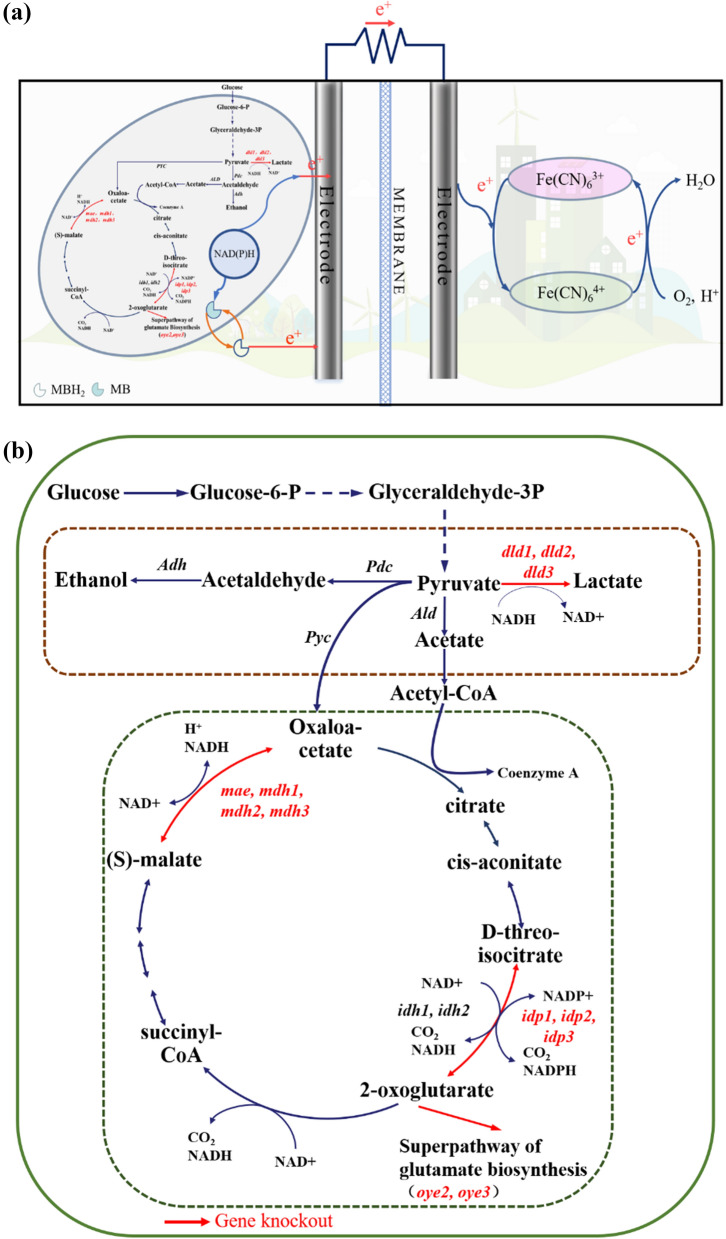
**a** Schematic diagram of yeast microbial fuel cell. **b** Regulation of the *Saccharomyces cerevisiae* metabolic pathway to accumulate excess electrons in the yeast cell. Knockdown of genes associated with NAD(P)H depletion in yeast cells (marked in red), specifically lactate dehydrogenase genes dld1, dld2, dld3 in the pyruvate metabolism and genes associated with depletion of reduced coenzymes in the citric acid cycle including mitochondrial malic enzyme mae, malate dehydrogenase mdh1, mdh2, mdh3, NADP-specific isocitrate dehydrogenases idp1, idp2, idp3, old yellow enzyme oye2, oye3.

In this work, we constructed a dual-chamber microbial fuel cell in which modified *Saccharomyces cerevisiae* in the anode chamber utilized glucose for ethanol production and transferred excess intracellular electrons—in the form of the electron carrier NAD(P)H—to the anode electrode via an electron transfer mediator, with the cathode K_3_[Fe(CN)_6_] acting as the electron acceptor. The regulation of intracellular metabolic pathways in *Saccharomyces cerevisiae* mainly targets two modules—the pyruvate metabolism and the citric acid cycle—by knocking down 12 genes that consume NAD(P)H intracellularly and regulating the intracellular NADH/NAD^+^ and NADPH/NADP^+^ ratios (Fig. 1), thus affecting the intracellular electron content and the extracellular electron transfer (EET) rate.

## Results

### Performance of ethanol and electricity co-production by knockout strains of lactate dehydrogenase in pyruvate metabolism

In *Saccharomyces cerevisiae* cells, lactate synthesis is a reduction pathway catalyzed by lactate dehydrogenase in the pyruvate metabolism, and this reduction process is accompanied by the oxidation of NADH to NAD^+^ (i.e., electron-consuming pathway). Therefore, knocking out the gene associated with lactate dehydrogenase enables the accumulation of electrons in the cell, which are then directed to the anode via an electron transfer mediator. Since the process of ethanol fermentation is accompanied by a large amount of NADH consumption [26], the NADH accumulated after knocking out the gene related to lactate dehydrogenase is also conducive to increase the ethanol production at the same time. To verify the performance of the modified strain for ethanol and electricity co-production using glucose in MFCs, the cell growth, ethanol yield, and electricity production performance were studied in an open circuit and an external circuit.

**Fig. 2:**
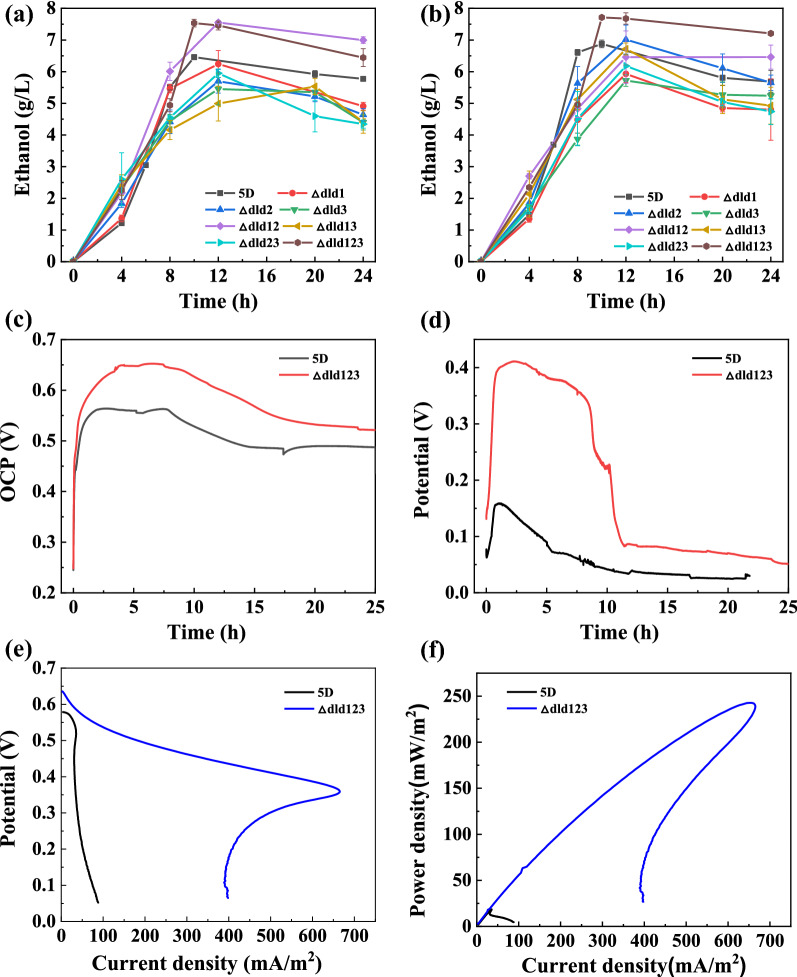
Ethanol and electricity co-production performances of knockout strains of lactate dehydrogenase. **a** Ethanol production yields of *5D*, *Δdld1*, *Δdld2*, *Δdld3*, *Δdld12*, *Δdld13*, *Δdld23*, and *Δdld123* knockout strains using glucose when run in the microbial fuel cell (MFC) for 24 h in an open circuit. **b** Ethanol production yields *5D* and seven knockout strains using glucose when run in the MFC for 24 h in an external circuit. **c** Open-circuit potential (OCP) of original *5D* strain and *Δdld123* strain running in the MFC for 24 h. **d** External-circuit potential of original *5D* strain and *Δdld123* strain operating in the MFC for 24 h with a 1000 Ω resistor connected externally in the MFC. **e** Linear scan voltammetry curves of the original *5D* strain and the *Δdld123* strain in the MFC at a sweep speed of 2 mv/s. **f** Power density curves of the original *5D* strain and the *Δdld123* strain in the MFC at a sweep speed of 2 mv/s. The MFC was run at 35 °C and 500 rpm. Values are shown as the mean ± s.d. (*n* = 3)

The growth curves of the strains (Additional file 1: Fig. S1) show that the OD of most of the knockout strains did not differ much from the growth of the original bacteria, so knocking out the lactate dehydrogenase-related genes in the pyruvate metabolism had little effect on the microbial growth. The knockout strains were examined for glucose consumption and ethanol production during the fermentation of ethanol using glucose in the open circuit and the e external circuit, respectively. The results showed that the highest yields of ethanol production from glucose fermentation by the *Δdld1*, *Δdld2*, *Δdld3*, *Δdld12*, *Δdld13*, and *Δdld23* strains reached 6.24, 7.01, 5.72, 7.55, 6.72, and 6.18 g/L, respectively (Fig. 2a, b). The *Δdld123* strain with all the lactate dehydrogenase genes knocked out had the highest ethanol yield of 7.71 g/L and the highest yield of 38.6% of all the knockout strains with external linkage resistance. The knockout strain consumed glucose at a similar rate to that of the original *5D* strain, with glucose being consumed at about 10 h. The specific values of the glucose consumption rate in the open circuit and the external circuit are shown in Additional file 1: Fig. S2. This indicates that knocking out all the relevant genes of lactate dehydrogenase does not affect ethanol production. This may be ascribed that knocking out lactate dehydrogenase leads to a greater flow of carbon sources into ethanol, resulting in a slight increase in ethanol production. However, it could also be due to the intracellular accumulation of more NADH, which increases the intracellular reducing power, resulting in an increase in ethanol production [26].

The MFC electrical production performance is shown in Fig. 2. The open-circuit potential of both the original and modified strains reached the highest value within the first 4 h. The highest open-circuit potential of the *5D* original bacteria reached 0.56 V. Figure 2c shows that the open-circuit potential of *Δdld123* strain was significantly increased compared with that of the original bacteria, reaching 0.65 V, which was 16% higher than that of the original bacteria. The highest open-circuit potentials of *Δdld1*, *Δdld2*, *Δdld3*, *Δdld12*, *Δdld13*, and *Δdld23* strains were 0.6, 0.586, 0.593, 0.584, 0.623, and 0.582 V, respectively (Additional file 1: Fig. S3), higher than the original open-circuit potential but lower than that of the *Δdld123* strain. The MFC showed a rapid decrease in the open-circuit potential for all strains after 10 h of operation, and the external circuit showed the same trend as the open circuit. The *5D* original strain had the highest external-circuit potential of 0.16 V, and among the other modified strains, the *Δdld12*3 strain had the highest external-circuit potential of 0.411 V, which was 157% greater than that of the original strain. However, the external circuit decayed more rapidly to only about 0.04 V at about 10 h. The reason for the decay of the open-circuit potential and the external-circuit potential may be that after the consumption of the substrate glucose, the electron supply is insufficient, resulting in the decay of the open-circuit potential and a corresponding decay of the external circuit. However, the decay of the external circuit is more rapid due to the consumption of electrons [27–29]. The greater improvement in the electricity production performance of the modified strain compared to that of the original *5D* strain may be due to the intracellular accumulation of more NADH after knocking out the gene related to lactate dehydrogenase. These accumulated electrons are in turn transferred to the electrode through the electron transfer medium methylene blue (MB), resulting in an increase in the open-circuit potential and external-circuit potential [26]. The performance of the microbial fuel cell was evaluated by linear scanning voltammetry at the open-circuit potential, and the results are shown in Fig. 2e, f. The results showed that the *Δdld123* strain had the highest power density, and the other knockout strains had power densities between 45 and 55 mW/m^2^, which were 2–3 times greater than that of the original bacteria. The highest power density of the *Δdld123* strain reached 240 mW/m^2^, which was 12 times greater than that of the original bacteria. The power density data for the remaining strains are shown in Additional file 1: Fig. S4. The large increase in the power density may be attributed to the accumulation of more electrons in the cell, which increases EET rate, resulting in a decrease in the internal resistance of the MFC [30], an increase in the external-circuit potential, and thus, an increase in the power density [31].

During the analysis of the power density curve, we observed the power overshoot phenomenon (Fig. 2e). The reasons for this phenomenon may be that by anode limitation, such as electron depletion phenomenon due to proton accumulation and substrate limitation, poor enrichment of biofilm, etc. [32, 33]. However, the power overshoot appears to be only a temporary system overload, as we observed that the microorganisms were able to overcome this overload during the ongoing polarization scan. Ieropoulos et al. proposed that during recovery, the electron/ion supply/demand balance is restored and the power profile exits the overload mode as the current starts to increase [34]. The recovery highlights the robustness of the microbial culture and its ability to adjust to dynamic and even hostile conditions, which can be attributed to the continuous replenishment of the depleted substrate [35].

### Performance of ethanol and electricity co-production by knockout strains of genes related to partial depletion of reduced coenzymes in the tricarboxylic acid cycle

After glucose is oxidized to pyruvate through the process of glycolysis, pyruvate is further oxidized to organic acids in the tricarboxylic acid cycle (TCA cycle). Since some pathways accumulate electrons in the TCA cycle [36], knockdown of some genes related to the consumption of reduced coenzymes in the TCA cycle (as marked by the TCA module in Fig. 1) is considered to result in the accumulation of electrons and improve the electricity production performance. The specific knockdown genes are shown in Table 1.

**Table 1:** Selected genes associated with the depletion of reduced coenzymes in the TCA cycle

Gene name	Pathway	Reaction	Dependency
*mae*	TCA cycle	(S)-malate + NAD^+^ → CO_2_ + pyruvate + NADH	NAD-dependent enzyme
*idp1, idp2, idp3*	Glutamate biosynthesis	D-threo-isocitrate + NADP^+^ $\leftrightarrow$ 2-oxoglutarate + CO_2_ + NADPH (reversible)	NADP-dependent enzyme
*mdh1, mdh2, mdh3*	TCA cycle	(*S*)-malate + NAD^+^ $\leftrightarrow$ oxaloacetate + NADH + H^+^ (reversible)	NAD-dependent enzyme
*oye2, oye3*	Sterol metabolism, citronellol synthesis	Oxidized electron acceptor + NADPH + H^+^ $\leftrightarrow$ reduced electron acceptor + NADP^+^(reversible)	NADPH-dependent enzyme

**Fig. 3:**
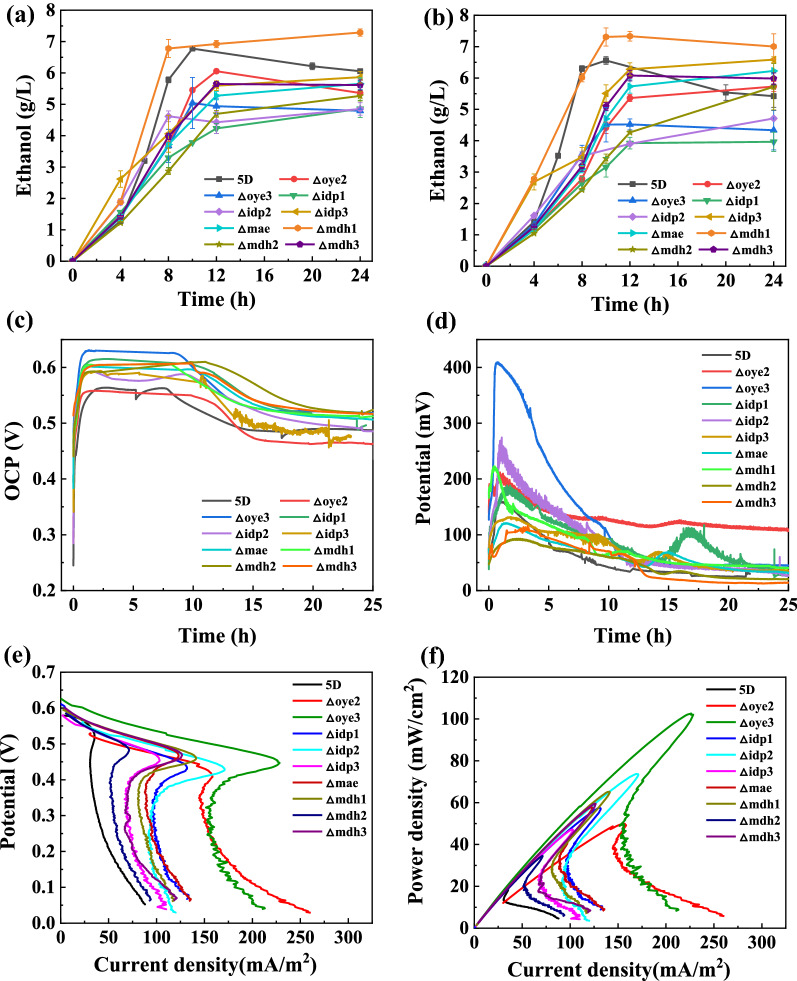
Ethanol and electricity co-production performances of knockout strains of genes associated with NAD(P)H consumption in the TCA cycle. **a** Ethanol production yields of *5D, Δmae, Δmdh1, Δmdh2, Δmdh3, Δidp1, Δidp2, Δidp3, Δoye2, and Δoye3* knockout strains using glucose when run in the MFC for 24 h with an open circuit. **b** Ethanol production yields *5D* and nine knockout strains using glucose when run in the MFC for 24 h in an external circuit. **c** Open-circuit potential of the original *5D* strain and nine knockout strains running in the MFC for 24 h. **d** External-circuit potential of the original *5D* strain and nine knockout strains operating in the MFC for 24 h with a 1000 Ω resistor connected externally in the MFC. **e** Linear scan voltammetry curves of the original *5D* strain and nine knockout strains in the MFC at a sweep speed of 2 mV/s. **f** Power density curves of the original *5D* strain and nine knockout strains in the MFC at a sweep speed of 2 mV/s. The MFC was run at 35 °C and 500 rpm. Values are shown as the mean ± s.d. (*n* = 3)

Knockout strains of genes associated with NAD(P)H consumption in the TCA cycle were similarly investigated for the ethanol electricity co-production performances of the knockout strains in terms of microbial growth, ethanol yield, and electricity production performance. The glucose consumption and ethanol production amounts of the knockout strains in the open circuit and the external circuit were examined separately. The ethanol production amounts of the knockout strains using glucose fermentation are shown in Fig. 3a, b. Aside from the highest ethanol production of 7.33 g/L for the·*Δmdh1* knockout strain, the ethanol production amounts of all the knockout strains decreased compared to that of the original *5D* strain. The glucose consumption rates of the knockout strains were lower, and there was glucose remaining in the anode electrolyte at 10 h (Additional file 1: Fig. S6). We observed from the strain growth curves (Additional file 1: Fig. S5) that most of the knockout strains had lower OD values than the original bacteria. The analysis of the reduction in ethanol yield after knockdown of some genes related to the depletion of reduced coenzymes in the TCA cycle may have occurred because the TCA cycle both provides energy for microbial life activities and is involved in the final metabolic pathway of the microorganism [37]. Therefore, the knockdown of the relevant genes in the TCA cycle will have an impact on microbial growth and nutrient metabolism, ultimately leading to a reduction in growth and ethanol yield.

The open-circuit and external-circuit potentials of the original bacteria and knockout strains are shown in Fig. 3c, d, respectively. The open-circuit potentials of the *Δoye3* and *Δidp1* knockout strains increased more significantly than those of other knockout strains, reaching 0.631 and 0.615 V, respectively, corresponding to increases of 12.7% and 9.8% compared with that of the original bacteria. A more significant increase in the external-circuit potential was observed for the *Δidp2* and *Δoye3* knockout strains, which reached 0.274 and 0.409 V, respectively. The performance of the microbial fuel cell was evaluated by linear voltammetry scanning, and the results showed that the highest power density of the *△oye3* strain reached 103.6 mW/m^2^, which was 5.6 times higher than that of the original bacteria. Most of the other knockout strains had power densities between 50 and 65 mW/m^2^, which were about three times higher than that of the original bacteria (Fig. 3e, f). Compared to the original *5D* strain, the strains that knocked out some of the genes related to the consumption of reduced coenzymes in the TCA cycle showed a slight improvement in electrical production, indicating that the *Δoye3* and *Δidp1* knockout strains were able to accumulate electrons intracellularly, which led to an increase in the open-circuit and external-circuit potentials and power densities [30]. Power overshoot was also observed in knockout strains of genes associated with NAD(P)H depletion in the TCA cycle.

### Performance of ethanol and electricity co-production of multi-knockout strains

The intracellular accumulation of electrons by adjusting two modules of the pyruvate metabolism and the TCA cycle improved the electricity generation performances of some of the knockout strains. Based on the above work, we chose to knock out the gene for electron accumulation in the TCA cycle based on the best performing *Δdld123* knockout strain for further electron accumulation.

**Fig. 4:**
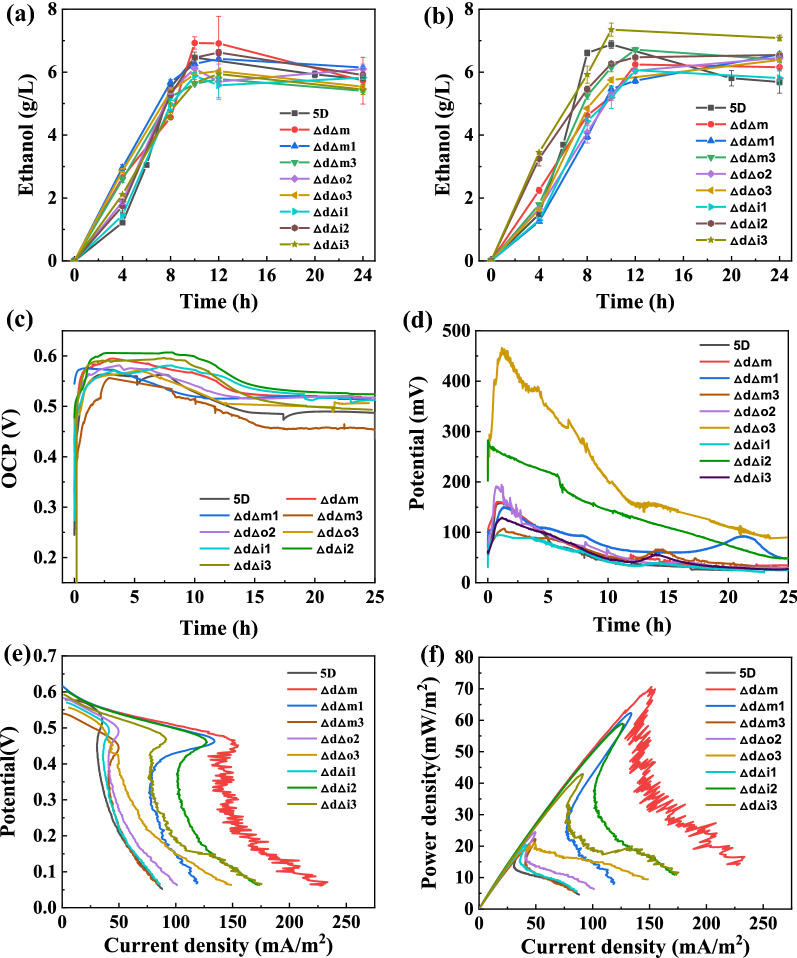
Performances of multiple knockout strains for ethanol and electricity co-production. **a** Ethanol production yields of *5D, ΔdΔm*, *ΔdΔm1*, *ΔdΔm3*, *ΔdΔi1*, *ΔdΔi2*, *ΔdΔi3*, *ΔdΔo2*, and *ΔdΔo3* knockout strains using glucose when run in the MFC for 24 h in an open circuit. **b** Ethanol production yields of *5D* and eight knockout strains using glucose when run in the MFC for 24 h in an external circuit. **c** Open-circuit potentials of the original *5D* strain and eight knockout strains running in the MFC for 24 h. **d** External-circuit potentials of the original *5D* strain and eight knockout strains operating in the MFC for 24 h with 1000 Ω resistors connected externally in the MFC. **e** Linear scan voltammetry curves of the original *5D* strain and eight knockout strains in the MFC at a sweep speed of 2 mV/s. **f** Power density curves of the original *5D* strain and eight knockout strains in the MFC at a sweep speed of 2 mV/s. The MFC was operated at 35 °C and 500 rpm. Values are shown as the mean ± s.d. (*n* = 3)

The ethanol yields of the knockout strains in the open and external circuits are shown in Fig. 4a, b, respectively. The ethanol yields of each of the multiple knockout strains were essentially the same as those of the original *5D* strain, and the glucose was consumed in the anode electrolyte at 10 h (Additional file 1: Fig. S8). Since knocking out some genes in the TCA cycle would affect the growth and metabolism of the strain, knocking out the above genes in the TCA cycle on top of the *Δdld123* knockout strain resulted in the majority of knockout strains having growth amounts that were still lower than that of the original bacteria (Additional file 1: Fig. S7). The reason for the absence of significant changes in ethanol production in the multiple knockout strains may be attributed to the combined action of two modules of the pyruvate metabolism and the TCA cycle.

The open-circuit and external-circuit potentials are shown in Fig. 4c, d, respectively, for the original strain and the multiple knockout strains. The open-circuit potential of the *ΔdΔi1* knockout strain increased significantly compared to those of the other knockout strains, reaching 0.615 V, and the external-circuit potential reached a maximum of 0.283 V. The open-circuit potential increased by 34 mV, and the external-circuit potential increased by 77% compared to those of the original strain. However, the electricity generation performance was inferior to that of the *Δdld123* with the knockdown of lactate dehydrogenase. The power densities of multiple knockout strains *ΔdΔm, Δd3Δm1,* and *Δd3Δi2* were 70.6, 62.3, and 59 mW/m^2^, respectively, which were 2–3 times higher than those of the original strain, and the rest of the strains did not differ much from the original strain. Similarly, we observed a power overshoot in the power density curves of the multiple knockout strains. The reason for the lack of a significant effect of multiple knockout strains on improving the electricity generation performance may be that multiple knockouts have an effect on the intracellular metabolism of the strains and the accumulated NAD(P)H acts on other metabolic pathways, leading to a re-consumption of NAD(P)H, which in turn leads to a decrease in power generation performance[38, 39].

### Intracellular electron accumulation

**Fig. 5:**
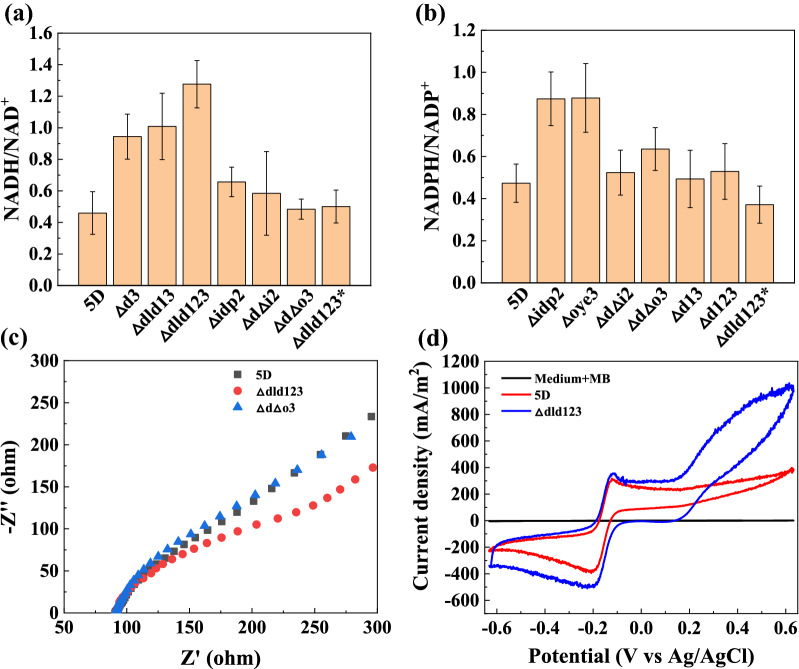
Diagram of intracellular electron accumulation verification. **a** Intracellular NADH/NAD^+^ ratios of *5D and Δd3*, *Δd13*, *Δd123*, *Δidp2*, *ΔdΔi2*, *ΔdΔo3*, and Δdld123* knockout strains cultured at 30 °C and 200 rpm in a shaker (“Δdld123*” indicates the *Δdld123* knockout strain cultured in the MFC). **b** Intracellular NADPH/NADP^+^ ratio of *5D and Δidp2*, *Δoye3*, *ΔdΔi2*, *ΔdΔo3*, *Δd13*, *Δd123*, and Δdld123* knockout strains Incubated in a shaker at 30 °C and 200 rpm. **c** Electrochemical impedance spectra of *5D*, *Δdld123*, and *ΔdΔo3* strains in a three-electrode system in the MFC. **d** Cyclic voltammetry curves of the blank medium and strains *5D* and *Δdld123* in the MFC at a sweep speed of 1 mV/s in the three-electrode system. The MFC was run at 35 °C and 500 rpm. Values are shown as the mean ± s.d. (*n* = 3)

According to our hypothesis, after the knockdown of electron-consuming genes, the intracellular electron consumption pathway was blocked and the intracellular electron accumulation increased. When the intracellular levels of total NAD (NADH + NAD^+^) and NADP (NADPH + NADP^+^) were kept constant, the increase in the intracellular releasable electrons can be presented by the NAD(P)H/NAD(P)^+^ ratio [26]. For this reason, we selected some knockout strains and cultured them in shake flasks, which were shaken at 30 °C and 200 rpm to measure NADH/NAD^+^ and NADPH/NADP^+^ ratios. The NADH/NAD^+^ ratio of the *Δdld123* knockout strain was 2.8 times higher than that of the original *5D* strain, and the ratio of the other knockout strains also showed varying degrees of increase (Fig. 5a). These results are consistent with our hypothesis that knockdown of lactate dehydrogenase significantly increases the number of releasable electrons intracellularly in the form of an increased NADH content. In contrast, the number of intracellular electrons is also increased to some extent in strains knocking out some genes of the TCA cycle, but not to the same extent as in the *Δdld123* knockout strain. As shown in Fig. 5(b), the intracellular NADPH/NADP^+^ values, which were 1.87 and 1.85 times higher for the *Δoye3* and *Δidp2* knockout strains than that of the original 5D strain, respectively. This indicates that knocking out the relevant genes from the strains in the TCA cycle mainly increases the intracellular electron accumulation in the form of an increased intracellular NADPH content. However, the increase in the intracellular NADPH content is not significant for the strains with multiple knockouts and knockout of lactate dehydrogenase. By measuring the intracellular NAD(P)H/NAD(P)^+^ ratio in the presence of electron transfer mediators in the MFCs, we observed the intracellular electron accumulation in the Δdld123* knockout strain was close to that of the original *5D* strain (Fig. 5a, b). This is because intracellular electrons are transferred from the cell to the electrode by extracellular electron transfer mediators, so the intracellular electron content of the *Δdld123* knockout strain decreases to the same level as that of the original *5D* strain, ultimately leading to a similar NAD(P)H/NAD(P)^+^ ratio in both strains. These results suggest that the electrons accumulated in the cells of the knockout strain are transferred to the anode of the MFC. It can also be speculated that the change in ethanol and electricity co-production performance of the multiple knockout strains may be due to a decrease in total intracellular NAD(P)H content from the change in total intracellular NAD(P)H.

The internal resistance of the knockout strain on the anode was assessed by measuring the electrochemical impedance spectrum (EIS) [40] to further determine the charge-transfer internal resistance and diffusion internal resistance of the MFC [30]. Here, the original strain and the *Δdld123* and *ΔdΔo3* knockout strains with higher outer circuits were selected. The electrochemical impedance spectra of all three strains are composed of a semicircle in the front and a straight line in the back. The *Δdld123* strain has a distinct semicircle part, and the other two strains have less evident semicircle parts, but the diameter of the semicircle part of the *Δdld123* strain is significantly smaller than that of the original strain, as shown in Fig. 5c. Since the semicircles of the EIS are not well-defined, the impedance data were fitted with the Randles equivalent circuit (Additional file 1: Fig. S9) to obtain accurate results, The charge-transfer internal resistance (R_ct_) of the *Δdld123* strain is 90 Ω, which is much smaller than that (149 Ω) of the *5D* strain, clearly indicating that the charge-transfer internal resistance of the *Δdld123* strain was reduced and the EET rate was significantly increased. Therefore, knockdown of intracellular electron-consuming genes can effectively facilitate EET efficiency in *Saccharomyces cerevisiae.* By comparing with other studies, the charge-transfer internal resistance in the microbial fuel cell mentioned in this study is much lower, showing even better performance (Table 2).

**Table 2:** Total internal resistance/charge-transfer internal resistance of different microbial fuel cells under the same or similar conditions

Types of microbial fuel cell anode microorganisms	Internal resistance (Ω)	Previous studies	References
Anaerobic activated sludge	82.1(R_ct_)	Yuan et al. 2011	[42]
*Escherichia coli*	134(R_ct_)	Liu et al. 2012	[30]
Mixed bacteria screened from marine surface sediments	34(R_ct_)	Du et al. 2015	[43]
Soil microbial	183	Li et al. 2016	[44]
The effluent from existing MFCs during operation	187	Kim et al. 2021	[32]
*Saccharomyces cerevisiae*	90(R_ct_)	–	This Study

As shown in Fig. 5d, the cyclic voltammetry curves with and without *Saccharomyces cerevisiae* in the MFC, from which it can be seen that there is no current response when the anode electrolyte is medium without *Saccharomyces cerevisiae* (Additional file 1: Fig. S10), while the current response is significant when *Saccharomyces cerevisiae* is added, and *Δdld123* has a larger current response than the original *5D* strain. In addition, two distinct redox peaks in the range of − 0.1 V to  − 0.2 V are present, indicating that *Saccharomyces cerevisiae* has a distinct biocatalytic behavior. In other words, it uses glucose for oxidation reactions at the anode to provide electrons and knocking out the dld gene significantly increases the electrochemical activity. The internal area of the cyclic voltammogram of the *Δdld123* knockout strain is significantly larger than that of the original *5D* strain, indicating a higher anodic conductance [41], i.e., a faster electron transfer rate, which is also mutually verified with the EIS images (Fig. 5c).

### Calculation of energy utilization

Using *Saccharomyces cerevisiae* in the MFC for ethanol and electricity co-production, part of the energy from glucose is used for fermentation to produce ethanol without affecting the yeast growth; part of the energy can be used for electricity generation by extracting electrons generated from the NADH/NAD^+^ redox cycle using MFC technology. Thus, part of the energy originally converted to heat by the microorganisms during fermentation can be collected in the form of electricity and the utilization rate of the substrate will be improved. For this process, we calculated the energy utilization of each knockout strain in an open circuit. We used the heat of combustion values to calculate the utilization of the substrate, with an equal scaling of the MFC scale by a factor of 10. The fermentation energy utilization is calculated as

1 $$\begin{gathered} \eta_{{1}} = {{n_{{{\text{eth}}}} *{\Delta H}_{{{\text{eth}}}} } \mathord{\left/ {\vphantom {{n_{{{\text{eth}}}} *{\Delta H}_{{{\text{eth}}}} } {n_{{{\text{glu}}}} *\Delta {\text{H}}_{{{\text{glu}}}} }}} \right. \kern-\nulldelimiterspace} {n_{{{\text{glu}}}} *\Delta {\text{H}}_{{{\text{glu}}}} }}, \hfill \\ \hfill \\ \end{gathered}$$

where *n*_eth_ and *n*_glu_ are the amounts of ethanol and glucose in the fermentation process, respectively, and *Δ*H_eth_ and *Δ*H_glu_ are the heats of combustion of ethanol and glucose, respectively.

The energy utilization during electricity generation is calculated with the following assumptions: the operation is carried out for 24 h at maximum power density, the volume scale is enlarged 10 times to 1 L, and the electrode size is enlarged 10 times in equal proportion. The energy utilization of power generation is defined as:

2 $$\begin{gathered} \eta_{{2}} = \, {{P*S*t} \mathord{\left/ {\vphantom {{P*S*t} {n_{{{\text{glu}}}} *\Delta {\text{H}}_{{{\text{glu}}}} }}} \right. \kern-\nulldelimiterspace} {n_{{{\text{glu}}}} *\Delta {\text{H}}_{{{\text{glu}}}} }}, \hfill \\ \hfill \\ \hfill \\ \end{gathered}$$

where *P* is the MFC power density, *S* is the electrode area, and *t* is the MFC operation time.

As shown in Table 3, the substrate energy utilization of some knockout strains is improved to some extent, with the *Δdld123* knockout strain increasing by 17.3% compared to that of the original strain. This indicates that the utilization of the substrate glucose can be improved by modifying *Saccharomyces cerevisiae* and applying it to the MFC.

**Table 3:** Summary of energy utilization values of knockout strains

Strains	Fermentation energy utilization (%)	Electricity generation energy utilization (%)	Total energy utilization (%)
*5D*	61.6	0.5	62.1
*Δdld1*	57.9	1.3	59.2
*Δdld2*	54.4	1.5	55.8
*Δdld3*	52.0	1.2	53.2
*Δdld12*	67.2	1.4	68.6
*Δdld13*	52.8	1.1	53.8
*Δdld23*	56.7	1.0	57.7
*Δdld123*	71.8	7.6	79.4
*Δoye2*	57.8	1.5	59.2
*Δoye3*	48.1	2.9	51.0
*Δidp1*	46.3	1.6	47.9
*Δidp2*	46.6	2.1	48.7
*Δidp3*	60.9	1.7	62.6
*Δmae*	53.9	1.7	55.6
*Δmdh1*	69.5	2.0	71.5
*Δmdh2*	50.2	1.0	51.3
*Δmdh3*	53.9	1.7	55.5
*ΔdΔo2*	61.8	0.7	62.5
*ΔdΔo3*	61.5	0.6	62.1
*ΔdΔi1*	58.3	0.6	58.8
*ΔdΔi2*	63.1	1.7	64.9
*ΔdΔi3*	70.1	1.3	71.4
*ΔdΔm*	66.0	1.8	67.9
*ΔdΔm1*	61.2	2.0	63.2
*ΔdΔm3*	63.1	0.0	63.2

## Discussion

The low power of MFCs is currently the main constraint for MFC applications [45]. The electroactive microorganisms in MFCs are mainly electroactive bacteria, such as *Geobacter sulfurreducens *[46], *Shewanella oneidensis MR-1 *[47], archaea [48], and eukaryotic microorganisms [21]. Current research on electroactive microorganisms also includes the modification of new genetically engineered microorganisms, such as *E. coli *[49] and *Saccharomyces cerevisiae *[50], to provide the MFC with more efficient electron transfer and increase the EET. There are currently two main strategies to improve MFC performance. One is to increase the EET rate, for example, by improving the electrode materials [51–53] or microbial co-culture to secrete extracellular polymers (e.g., riboflavin) [9, 54], and the other is to increase intracellular electron accumulation to increase the electron supply. In microbial cells, increasing the supply of electrons can be achieved by various methods, including introducing exogenous genes that enhance intracellular NADH regeneration [36, 55], targeting metabolic fluxes for NAD^+^ biosynthesis using modular engineering [56, 57], and knocking down intracellular reductive metabolic pathways [26]. Knocking out of lactate synthesis pathway genes [26] and central metabolism genes [38] in *E. coli* increases the intracellularly releasable electrons, which are subsequently transferred to the anode via an electron transfer medium, improving the MFC electrical production performance.

*Saccharomyces cerevisiae* is a microorganism suitable for industrial production, and its fermentation of glucose for ethanol production is accompanied by the oxidation of large amounts of NADH to NAD^+^ (electron consumption pathway) [58, 59]. This property of *Saccharomyces cerevisiae* makes it an ideal anode microbial catalyst in microbial fuel cells. For the improvement of yeast microbial fuel cell performance, Manisha Verma et al. summarized that the current methods are addition of artificial mediators, anode surface modification, yeast cell immobilization, yeast surface display method, genetically modified yeast cell, etc. [60].

Here, we enhance the performance of MFC by modulating in vivo metabolism in *Saccharomyces cerevisiae*. Knockdown of genes involved in the depletion of NAD(P)H allows intracellular accumulation of reduced coenzymes (i.e., electrons).

## Conclusion

In this study, it was found that the knockdown of intracellular electron-consuming genes allowed the accumulation of excess electrons in cells, and the elevated intracellular electron pool positively influenced the electrical production performance of the MFC. Furthermore, by knocking out the intracellular metabolic pathway, the yield of ethanol could be increased, and co-production of ethanol and electricity could be achieved. Thus, the MFC improved the utilization of the substrate. The highest ethanol yield of the knockout strain reached 7.71 g/L, and the highest power density in the MFC reached 240 mW/m^2^, which was 12 times higher than that of the control bacteria. In addition, the energy utilization was 17.3% higher than that of the control bacteria. Since there are many electron-consuming and electron-generating pathways in *Saccharomyces cerevisiae*, our work explored the reduction of electron consumption. We will subsequently consider enhancing the electron-generating pathways to enhance intracellular electron accumulation and further improve the electricity production performance. In addition, enhancements in terms of ethanol production, such as improving carbon utilization through CO_2_ reuse [61], can be considered. This work provides a reference for subsequent in vivo metabolic engineering of microorganisms to regulate intracellular electron accumulation for bioelectricity production in MFCs.

## Methods

### Strain construction

The *Saccharomyces cerevisiae* used in the experiments had the conservation number CEN.PK 117-5D. The lactate dehydrogenase genes dld1, dld2, and dld3 in the pyruvate metabolism were knocked out using CRISPR/Cas9. The same approach was used to knock out some of the genes related to the consumption of reduced coenzymes in the citric acid cycle, including mae, mdh1, mdh2, mdh3, idp1, idp2, idp3, oye2, and oye3. Additional file 1: Table S1 and Sect. 1.1 of the additional file information list the specific primers and methods of operation.

### Preparation of cathode and anode chambers

For the cathode electrolyte, 0.5 g of potassium ferricyanide (Macklin biochemical Co., Ltd, China, Shanghai) was dissolved in 100 ml of phosphate buffer solution (PBS) with a pH of 6.5 to obtain a 5 g/L solution. The anode electrolyte contained basal medium (7.5 g/L NH_4_SO_4_ (Macklin biochemical Co., Ltd, China, Shanghai), 14.4 g/L KH_2_PO_4_ (Aladdin Biochemical Technology Co., Ltd, China, Shanghai), 0.5 g/L MgSO_4_·7H_2_O (Sinopharm Chemical Reagent Co., Ltd, China, Beijing), 0.1% vitamin solution, and 0.1% trace metal solution [62]). The anode electrolyte was adjusted to a pH of 6.5 with KOH and autoclaved at 116 °C for 25 min.

### Construction of microbial fuel cells

The microbial fuel cell device was a typical H-type microbial fuel cell, consisting of two glass bottles with volumes of 100 ml. The cathode and anode chambers were separated with Nafion 117 membranes (DuPont, USA, State of Delaware). Carbon cloth was used as the electrodes with anode electrode sizes of 2.5 × 3 cm and cathode electrode sizes of 3 × 3 cm. Carbon cloth electrodes and proton exchange membranes were treated (Additional file 1: Sect. 1.2), and the microbial fuel cell device was assembled in an ultraclean bench. Since oxygen is beneficial for the pre-growth of microorganisms, the anode electrolyte was not treated with N_2_ venting. A 1 kΩ resistor was connected externally for all the external-circuit potential measurements.

*Saccharomyces cerevisiae* strains were inoculated into yeast extract peptone dextrose (YPD) medium (20 g/L glucose (Sinopharm Chemical Reagent Co., Ltd, China, Beijing), 20 g/L peptone (Oxoid, UK), and 10 g/L yeast extract (Oxoid, UK)) and incubated at 30 °C and 200 rpm. Yeast cells were inoculated into the anode chamber after centrifugation and washing with an initial optical density (OD) of approximately 1.5. The microbial fuel cell device was placed on a magnetic hot plate at 35 °C with continuous agitation of the anode electrolyte at 500 rpm/min to ensure the growth of the anode microorganisms and the contact of the microorganisms with the electrodes. The sampling port was disinfected by spraying 75% ethanol before sampling, and samples were taken with sterile syringes.

### Analysis of products and electrochemistry

The biomass of the electricity generation process was determined by measuring the absorbance values of the samples at 600 nm using a UV spectrophotometer (OnLab, China, Shanghai). An LC20-AT high-performance liquid chromatograph (Shimadzu, Japan) was used to detect the concentration of glucose and ethanol in the anode electrolyte. A BIO-Rad 87H column was used with 5 mM sulfuric acid as the mobile phase, a column temperature of 65 °C, a flow rate of 0.6 ml/min, an injection volume of 10 μL, and a detector with an oscillometric detector. Intracellular NAD(P)^+^ and NAD(P)H were analyzed using NADH/ NAD^+^ and NADPH/ NADP^+^ quantification kit (Suzhou Comin Biotechnology Co., Ltd., USA). The open-circuit potential and external-circuit potential of the MFC were measured using a potentiostat (CHI660E, Chenhua Co., Ltd., China, Shanghai). The polarization curves were obtained using linear scanning voltammetry with a sweep rate of 2 mV/s. Cyclic voltammetry and electrochemical impedance spectroscopy (EIS) were conducted using a three-electrode system with Ag/AgCl as the reference electrode and a cyclic voltammetry scan rate of 1 mV/s. EIS were conducted over a frequency range of 0.01 Hz to 100 kHz at open circuit potential, with a perturbation signal of 10 mV. Nyquist plots were simulated as an equivalent circuit (Additional file 1: Fig. S9) using a fitting program (ZsimpWin 3.10).

## Supplementary Information

**Additional file 1: Figure. S1.**Growth curves of lactate dehydrogenase knockout strains in MFC run in (a)open circuit for 24 h; (b) external circuit for 24 h. **Figure. S2.**Glucose consumption curve of lactate dehydrogenase knockout strains in MFC run in (a)open circuit for 24 h; (b) external circuit for 24 h. **Figure. S3.**Open circuit voltage of lactate dehydrogenase knockout strains running in MFC for 24 h. **Figure. S4. **(a) Linear scanning voltammetry curves of the lactate dehydrogenase knockout strain in MFC at a sweep speed of 2 mv/s; (b) power density curves of the lactate dehydrogenase knockout strain in MFC at a sweep speed of 2 mv/s. **Figure. S5.**Growth curves of knockout strains associated with NAD(P)H depletion in the TCA cycle in MFC run in (a)open circuit for 24 h; (b) external circuit for 24 h. **Figure. S6.**Glucose consumption curve of knockout strains associated with NAD(P)H depletion in the TCA cycle in MFC run in (a)open circuit for 24 h; (b) external circuit for 24 h. **Figure. S7.**Growth curves of multi-knockout strains in MFC run in (a)open circuit for 24 h; (b) external circuit for 24 h. **Figure. S8.**Glucose consumption curve of multi-knockout strains in MFC run in (a)open circuit for 24 h; (b) external circuit for 24 h. **Figure. S9** The schematic of an equivalent circuit model(R(Q(RW))). **Figure. S10.**Cyclic voltammetry curve of the blank control at a sweep speed of 1 mV/s. **Table S1.** Relevant primers for constructing knockout plasmids (genes dld1, dld2, dld3, mae, mdh1, mdh2, mdh3, oye2, oye3, idp1, idp2, idp3). **Table S2.** Construction of knockout plasmids for donor (genes dld1, dld2, dld3, mae, mdh1, mdh2, mdh3, oye2, oye3, idp1, idp2, idp3). **Table S3.** Name of strains used in this study. **Table S4.** Summary of the highest ethanol yields for each knockout strain.

## Data Availability

All data generated or analyzed during this study are included in this published article and its additional files.

## Abbreviations

MFCs: Microbial fuel cells
DET: Direct electron transfer
EET: Extracellular electron transfer
OCP: Open-circuit potential
MB: Methylene blue
TCA cycle: Tricarboxylic acid cycle
EIS: Electrochemical impedance spectroscopy
