# Supplementary material for: Improved energy efficiency in microbial fuel cells by bioethanol and electricity co-generation
Source: Biotechnol Biofuels Bioprod. 2022 Aug 17;15:84. doi: 10.1186/s13068-022-02180-4 (PMC9382818; doi:10.1186/s13068-022-02180-4)
Supplement: Supplementary file 1 — Additional file 1: Figure. S1.Growth curves of lactate dehydrogenase knockout strains in MFC run in (a)open circuit for 24 h; (b) external circuit for 24 h. Figure. S2.Glucose consumption curve of lactate dehydrogenase knockout strains in MFC run in (a)open circuit for 24 h; (b) external circuit for 24 h. Figure. S3.Open circuit voltage of lactate dehydrogenase knockout strains running in MFC for 24 h. Figure. S4. (a) Linear scanning voltammetry curves of the lactate dehydrogenase knockout strain in MFC at a sweep speed of 2 mv/s; (b) power density curves of the lactate dehydrogenase knockout strain in MFC at a sweep speed of 2 mv/s. Figure. S5.Growth curves of knockout strains associated with NAD(P)H depletion in the TCA cycle in MFC run in (a)open circuit for 24 h; (b) external circuit for 24 h. Figure. S6.Glucose consumption curve of knockout strains associated with NAD(P)H depletion in the TCA cycle in MFC run in (a)open circuit for 24 h; (b) external circuit for 24 h. Figure. S7.Growth curves of multi-knockout strains in MFC run in (a)open circuit for 24 h; (b) external circuit for 24 h. Figure. S8.Glucose consumption curve of multi-knockout strains in MFC run in (a)open circuit for 24 h; (b) external circuit for 24 h. Figure. S9 The schematic of an equivalent circuit model(R(Q(RW))). Figure. S10.Cyclic voltammetry curve of the blank control at a sweep speed of 1 mV/s. Table S1. Relevant primers for constructing knockout plasmids (genes dld1, dld2, dld3, mae, mdh1, mdh2, mdh3, oye2, oye3, idp1, idp2, idp3). Table S2. Construction of knockout plasmids for donor (genes dld1, dld2, dld3, mae, mdh1, mdh2, mdh3, oye2, oye3, idp1, idp2, idp3). Table S3. Name of strains used in this study. Table S4. Summary of the highest ethanol yields for each knockout strain. [file 13068_2022_2180_MOESM1_ESM.docx]

**Additional Information**

**Improved energy efficiency in microbial fuel cells by** **bioethanol and electricity co-generation**

Rong Xie ^a^, Shuang Wang ^a^, Kai Wang ^a^, Meng Wang ^a^, Biqiang Chen, Zheng Wang ^a^, Tianwei Tan ^a*^

^a^National Energy R&D Center for Biorefnery, Beijing Key Lab of Bioprocess, College of Life Science and Technology, Beijing University of Chemical Technology, No. 15 North 3rd Ring Rd East, 100029 Beijing, PR China.

*Correspondences:twtan@mail.buct.edu.cn

E-mail address: College of Life Science and Technology, Beijing University of Chemical Technology, No. 15 North 3rd Ring Rd East, 100029 Beijing, PR China.

**Experimental method**

1.1 Strain construction

(1) Construction of lactate dehydrogenase knockout plasmids

The gRNAs required for gene knockdown were designed through the website (https://www.atum.bio/eCommerce/cas9/input). gRNAs were designed on primers, and fragments gRNA-dld1, gRNA-dld2, gRNA with uracil (Ura) screening marker were amplified using the Pstg.Ura plasmid as template. These three fragments were sequentially ligated to plasmid pLacZ-SalI and sequenced to obtain the correct plasmids pCas9-dld1, pCas9-dld2, and pCas9-dld3. Subsequently, the knockdown of each gene was performed according to the traditional Cas9 knockdown method for Saccharomyces cerevisiae genes. The primers and donor used in the experiments are shown in Table S1 and Table S2.

(2) Construction of relevant knockout plasmids in the TCA cycle

The correct sequenced plasmids pCas9- mae, pCas9-mdh1, pCas9-mdh2, pCas9-mdh3, pCas9-idp1, pCas9-idp2, pCas9-idp3, pCas9-oye2, pCas9-oye3 were obtained according to the same method as above. The primers and donor are shown in Table S1 and Table S2.

1.2 The construction of MFC

(1) Electrode pretreatment: carbon cloth electrode anode size is 2.5*3cm, cathode size is 2.5*2.5cm. The cut carbon cloth is soaked in 1 M HCl solution overnight, rinsed repeatedly in distilled water, soaked in acetone overnight and then removed and dried. The electrodes can be sterilized directly with the electrolytic cell.

(2) Proton exchange membrane: soak in 1M HCl solution overnight, rinse with sterilized water and then sterilize with UV lamp in a clean bench.

(3) The microbial fuel cell device was assembled in a clean bench. The proton membrane was cleaned with sterile distilled water and clamped for separating the anode and cathode chambers. The cathode and anode electrolytes were added to the device separately, and 250 μl of methylene blue solution was added to the anode chamber as an electron transfer mediator

**Results**

Fig. S1 Growth curves of lactate dehydrogenase knockout strains in MFC run in (a)open circuit for 24 h; (b) external circuit for 24 h

Fig. S2 Glucose consumption curve of lactate dehydrogenase knockout strains in MFC run in (a)open circuit for 24 h; (b) external circuit for 24 h

Fig. S3 Open circuit voltage of lactate dehydrogenase knockout strains running in MFC for 24 h.

Fig. S4 (a) Linear scanning voltammetry curves of the lactate dehydrogenase knockout strain in MFC at a sweep speed of 2 mv/s; (b) power density curves of the lactate dehydrogenase knockout strain in MFC at a sweep speed of 2 mv/s.

Fig. S5 Growth curves of knockout strains associated with NAD(P)H depletion in the TCA cycle in MFC run in (a)open circuit for 24 h; (b) external circuit for 24 h

Fig. S6 Glucose consumption curve of knockout strains associated with NAD(P)H depletion in the TCA cycle in MFC run in (a)open circuit for 24 h; (b) external circuit for 24 h

Fig. S7 Growth curves of multi-knockout strains in MFC run in (a)open circuit for 24 h; (b) external circuit for 24 h

Fig. S8 Glucose consumption curve of multi-knockout strains in MFC run in (a)open circuit for 24 h; (b) external circuit for 24 h.


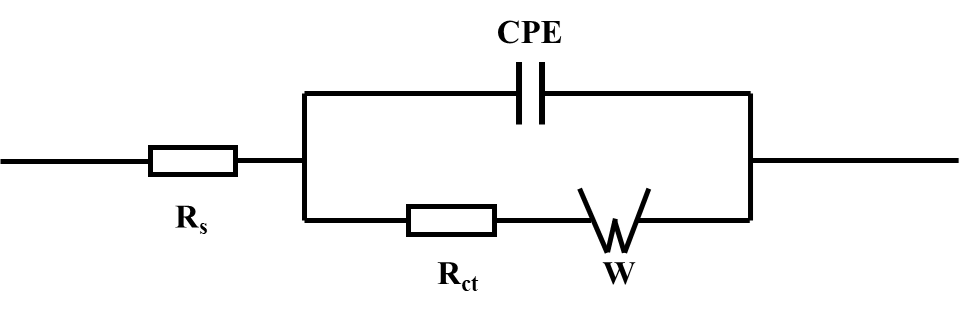


Fig. S9 The schematic of an equivalent circuit model(R(Q(RW))).

Fig. S10 Cyclic voltammetry curve of the blank control at a sweep speed of 1 mV/s

Table S1 Relevant primers for constructing knockout plasmids (genes dld1, dld2, dld3, mae, mdh1, mdh2, mdh3, oye2, oye3, idp1, idp2, idp3)

| Primer name | Gene sequence (5' to 3') |
| --- | --- |
| DLD1-Cas9-F | AAAGGTCTCTGATCAAGACCCAGAAAGTCGAGCGCGTTTTAGAGCTAGA |
| DLD1-Cas9-R | AAAGGTCTCTAAACTATTCTTGTAGGCAGGAAGTGATCATTTATCTTTCA |
| DLD2-Cas9-F | AAAGGTCTCTGATCATATATTCTTATCGAAACGTCGTTTTAGAGCTAGAA |
| DLD2-Cas9-R | AAAGGTCTCTAAACTTTAGGATCATAATGAACCTTGATCATTTATCTTTC |
| DLD3-Cas9-F | AAAGGTCTCTGATCAAAATTTCAAAGTTCTCGACTGTTTTAGAGCTAGA |
| DLD3-Cas9-R | AAAGGTCTCTAAACTGTATTGTCCTTCCTTAGGGCGATCATTTATCTTTC |
| MAE-CAS9-F | AAAGGTCTCTGATCAAACAATTGTTACTCTTTCCCGTTTTAGAGCTAG |
| MAE-CAS9-R | AAAGGTCTCTAAACTCCTCGAGTGCTTGCAAGATCGATCATTTATCTT |
| MDH1-CAS9-F | AAAGGTCTCTGATCATTTGCATCTCCGGTCACTTTGTTTTAGAGCTAG |
| MDH1-CAS9-R | AAAGGTCTCTAAACTTTCCTGAGTTGGATCGGTGTGATCATTTATCTTT |
| MDH2-CAS9-F | AAAGGTCTCTGATCACCTGTAATTGGCGGGCATTCGTTTTAGAGCTAG |
| MDH2-CAS9-R | AAAGGTCTCTAAACTGCATTCACGTTAAATAAGTCGATCATTTATCTT |
| MDH3-CAS9-F | AAAGGTCTCTGATCAGATTTGTTCAAGATGAACGCGTTTTAGAGCTAG |
| MDH3-CAS9-R | AAAGGTCTCTAAACTATGCCTTCCGCAGCTCGGATGATCATTTATCTT |
| OYE2-CAS9-F | AAAGGTCTCTGATCACTTTCCCTCTCCACAATCTGGTTTTAGAGCTAG |
| OYE2-CAS9-R | AAAGGTCTCTAAACTTCCCTAGCAAGGGTGTCTGGGATCATTTATCTT |
| OYE3-CAS9-F | AAAGGTCTCTGATCAGAGTGGGCTGCTGTGTATTAGTTTTAGAGCTAG |
| OYE3-CAS9-R | AAAGGTCTCTAAACTCGGAATATTCTCCTTCGCCCGATCATTTATCTTT |
| IDP1-CAS9-F | AAAGGTCTCTGATCAACCATCAGAAACATTCTCGGGTTTTAGAGCTAG |
| IDP1-CAS9-R | AAAGGTCTCTAAACTCTGCCCTTGTAGTCATACACGATCATTTATCTTT |
| IDP2-CAS9-F | AAAGGTCTCTGATCAAGTTTGAATCCCTTGGCATCGTTTTAGAGCTAG |
| IDP2-CAS9-R | AAAGGTCTCTAAACTATGATGATGGGCTTCTCCCAGATCATTTATCTT |
| IDP3-CAS9-F | AAAGGTCTCTGATCATGCCACTATAACACCCGATGGTTTTAGAGCTAG |
| IDP3-CAS9-R | AAAGGTCTCTAAACTTGGGCAGCCTCGCTTTCAAAGATCATTTATCTT |

Table S2 construction of knockout plasmids for donor (genes dld1, dld2, dld3, mae, mdh1, mdh2, mdh3, oye2, oye3, idp1, idp2, idp3)

| Primer name | Gene sequence (5' to 3') |
| --- | --- |
| Donor-DLD1-F | TATAGTGCAATTTAAATATTGTACATCATTCCGATCCAGCTGGAAACAA |
| Donor-DLD1-R | ATATATTACATGAAATATATTTCAGGTTTACGTGAAGGGTGAAAAAG |
| Donor-DLD2-F | ACAACTTCTTCTCTTTATTTTACAGACATTAATCCAGCACTACAAGTGGC |
| Donor-DLD2-R | GAATATAATATTGATAAATATATACATATGTAGATAACTATAAAACTTG |
| Donor-DLD3-F | TGCTCCTCAATATTTTATTCATTTGAGATTCAAGGCTTAAAGACAGCATA |
| Donor-DLD3-R | AAATCTATAATACTGTAAAGAAAAAGGGTTTGCTCTTTGAAAGTTAAAA |
| Donor-MAE-F | CTACAACTTTTAACGAGTTTAGTGCACATAAATACCAAGACAAAAGGTA |
| Donor-MAE-R | TATTTATATATTACTCTATATGGTTTTTTTTTTTAAGTGCAGGCGTTGGTT |
| Donor-MDH1-F | CGTGGACATCTACGGAAAGGAAGAAAAAAAACAAAAGGAAAAGGAAG |
| Donor-MDH1-R | ATTAGTAGAATTTTTTTTTTTTTTTCCCTATTTTTCACTCTATTTCTGATCTT |
| Donor-MDH3-F | GAATATAGTGCTGCAGACTATTACAAAAGTTCAATACAATATCATAAAA |
| Donor-MDH2-R | GACTGGCTTAACGGGAATATTATCAATTTGCTGCATTCTTATGCTTCGGT |
| Donor-MDH2-F | AATCAAGGGAAAACACTTGTCAGTGCAAAAGAAAATAAAAAGAGACAA |
| Donor-MDH3-R | CGATATGAGTCAAGATACAAAGGAGTATAGAGTTAAGAAAAATATAAA |
| Donor-OYE2-F | TCCAGATATAGAATAAATCATCATATTAAGCTAAATATAGACGATAATA |
| Donor-OYE2-R | TTCATTAATTATATAAATTAGAAGAAAAAGAAATGGTGCTACAAAGTAC |
| Donor-OYE3-F | AGTACGTACTTGATATATACAACAACTGTAGTTCAGTATAGCGAAGTTT |
| Donor-OYE3-R | TAATTAAAAATATGGCAGGAATATGAAAAATACATAACATCAATGTCTT |
| Donor-IDP1-F | TTATGAAATCTTCCTTCAAGCAATTGTGAGACAACAGACGCACAAGGAA |
| Donor-IDP1-R | TAAGTATAACACTTTAAAAAATGAAAAAAAAAAGTGGTAGATTGGGCT |
| Donor-IDP2-F | GCTGCTCAGGCACGAGAATAGGAGGTAAGAAGGTAACGTACGTATATA |
| Donor-IDP2-R | AAGCTTTTCGATAAAAAGGGAATATATAATATAAATAAATCAATCTGTC |
| Donor-IDP3-F | CTCCTGCACATACCTGCAGTAAACACAAGCAACACTTTAGAGATAGTTG |
| Donor-IDP3-R | CAAGTCTGAACACTATAACCCTAGGCCAGACTTGTCTTTTCAAATGAAT |

Table S3 Name of strains used in this study

| Strain name | Knockout gene |
| --- | --- |
| *Δdld12* | *Δdld1Δdld2* |
| *Δdld13* | *Δdld1Δdld3* |
| *Δdld23* | *Δdld2Δdld3* |
| *Δdld123* | *Δdld1Δdld2Δdld3* |
| *ΔdΔo2* | *Δdld1Δdld2Δdld3Δoye2* |
| *ΔdΔo3* | *Δdld1Δdld2Δdld3Δoye3* |
| *ΔdΔi1* | *Δdld1Δdld2Δdld3Δidp1* |
| *ΔdΔi2* | *Δdld1Δdld2Δdld3Δidp2* |
| *ΔdΔi3* | *Δdld1Δdld2Δdld3Δidp3* |
| *ΔdΔm* | *Δdld1Δdld2Δdld3Δmae* |
| *ΔdΔm1* | *Δdld1Δdld2Δdld3Δmdh1* |
| *ΔdΔm3* | *Δdld1Δdld2Δdld3Δmdh3* |

Table S4 Summary of the highest ethanol yields for each knockout strain

| Strain | Ethanol yield (%) |
| --- | --- |
| *5D* | 35.5 |
| *Δdld1* | 30.3 |
| *Δdld2* | 35.1 |
| *Δdld3* | 28.6 |
| *Δdld12* | 35.2 |
| *Δdld13* | 33.6 |
| *Δdld23* | 30.9 |
| *Δdld123* | 38.6 |
| *Δoye2* | 30.3 |
| *Δoye3* | 25.2 |
| *Δidp1* | 24.3 |
| *Δidp2* | 24.5 |
| *Δidp3* | 32.4 |
| *Δmae* | 31.1 |
| *Δmdh1* | 36.7 |
| *Δmdh2* | 28.6 |
| *Δmdh3* | 30.4 |
| *ΔdΔo2* | 32.4 |
| *ΔdΔo3* | 32.2 |
| *ΔdΔi1* | 30.6 |
| *ΔdΔi2* | 33.1 |
| *ΔdΔi3* | 36.8 |
| *ΔdΔm* | 34.6 |
| *ΔdΔm1* | 32.1 |
| *ΔdΔm3* | 33.1 |
